# Supplementary material for: The Role of Electrical Polarity in Electrospinning and on the Mechanical and Structural Properties of As-Spun Fibers
Source: Materials (Basel). 2020 Sep 19;13(18):4169. doi: 10.3390/ma13184169 (PMC7560487; doi:10.3390/ma13184169)
Supplement: Supplementary file 1 [file materials-13-04169-s001.pdf]

materials

MDPI

Supplementary Materials

The Role of Electrical Polarity in Electrospinning and on the Mechanical and Structural Properties of As-Spun Fibers

Daniel P. Ura [1], Joan Rosell-Llompart [2,3], Angelika Zaszczyńska [4], Gleb Vasilyev [5], Arkadiusz Gradys [4], Piotr K. Szewczyk [1], Joanna Knapczyk-Korczak [1], Ron Avrahami [5], Alena O. Šišková [6], Arkadii Arinstein [5], Paweł Sajkiewicz [4], Eyal Zussman [5] and Urszula Stachewicz [1,*]

1 International Centre of Electron Microscopy for Materials Science, Faculty of Metals Engineering and Industrial Computer Science, AGH University of Science and Technology, 30-059 Kraków, Poland; urad@agh.edu.pl (D.P.U.); pszew@agh.edu.pl (P.K.S.); jknapczyk@agh.edu.pl (J.K.-K.);
2 Department of Chemical Engineering, Universitat Rovira i Virgili, Av. dels Països Catalans 26, 43007 Tarragona, Spain, joan.rosell@urv.cat
3 Catalan Institution for Research and Advanced Studies - ICREA, Pg. Lluís Companys 23, 08010 Barcelona, Spain
4 Laboratory of Polymers and Biomaterials, Institute of Fundamental Technological Research, Polish Academy of Sciences, 02-106 Warszawa, Poland; angelika.zaszczynska@gmail.com (A.Z.); arkadiuszgradys@gmail.com (A.G.), psajk@ippt.pan.pl (P.S.)
5 NanoEngineering Group, Faculty of Mechanical Engineering, Technion–Israel Institute of Technology, 32000 Haifa, Israel; mevasil@me.technion.ac.il (G.V.); ronavra@technion.ac.il (R.A.); mearin@technion.ac.il (A.A.); meeyal@technion.ac.il (E.Z.)
6 Polymer Institute of Slovak Academy of Sciences, 845 41 Bratislava, Slovakia; alena.siskova@savba.sk
* Correspondence: ustachew@agh.edu.pl; Tel.: +48-12-617-52-30

Received: 3 August 2020; Accepted: 17 September 2020; Published: date

In this supporting data file, we provide example time traces of the measurements of applied voltage on the needle and collected electrical current for positive and negative voltage polarity during electrospinning (Figure 1S), differential scanning calorimetry (DSC) curves for electrospun poly methyl methacrylate (PMMA) fibers (Figure 2S), and scanning electron microscopy (SEM) micrographs of cross-sectional electrospun mats used for mechanical testing (Figure 3S). Table 1S shows the average and standard deviation for the applied voltage and the collector current. Table 2S shows the glass transition temperature (T_g) at the first and the second heating runs, crystallinity and molecular mass for PMMA powder and electrospun random and aligned fibers, produced with positive and negative voltage polarity. Table 3S shown material parameters used for calculations of $\tilde{L}_E, \tilde{R}_E$, $\tilde{E}$ and q.

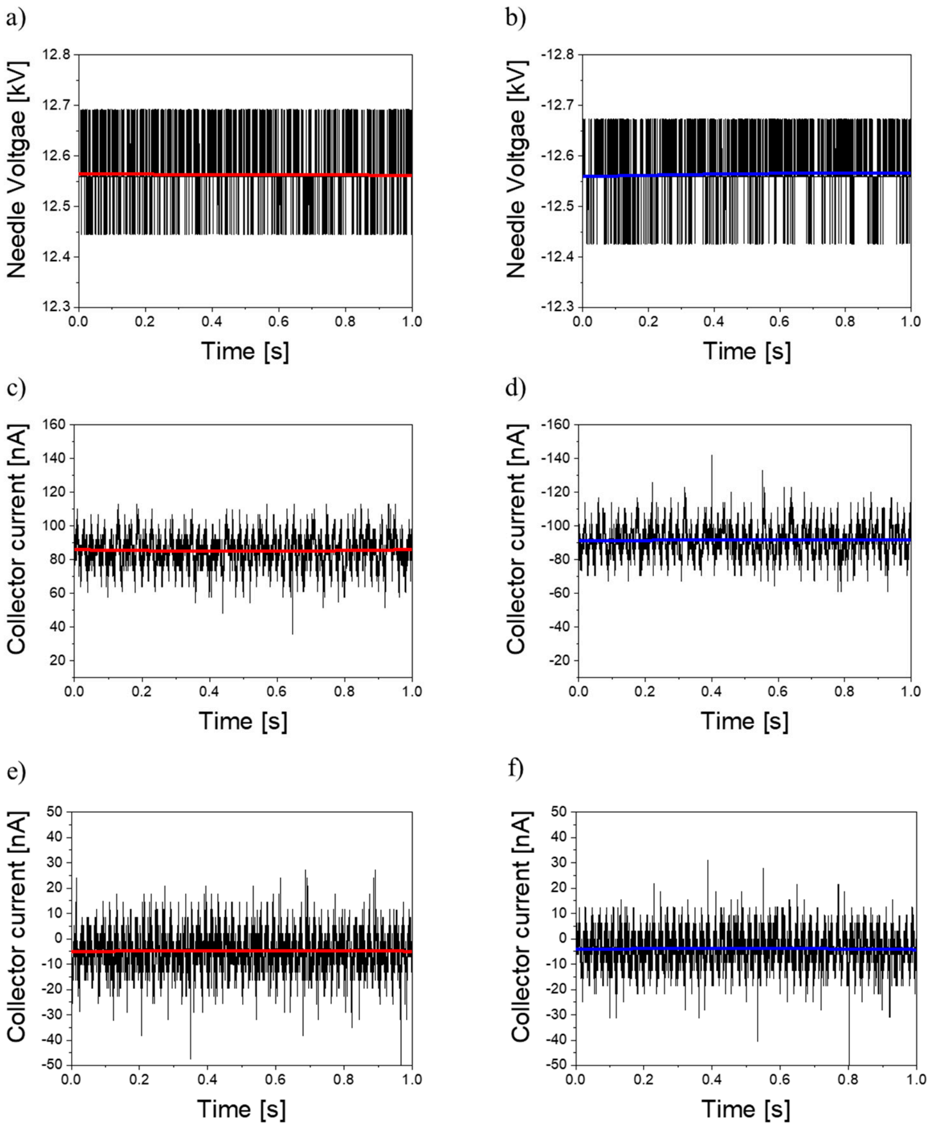

Figure S1. Examples of time traces of the needle voltage, (**a**) and (**b**), of the measured collector electrical current with a flow of polymer solution during electrospinning, (**c**) and (**d**), and of the measured collector electrical current baseline (the current without any flow of polymer solution), (**e**) and (**f**). The panels on the left are for the positive polarity, while the panels on the right are for negative polarity. The collector was a brass plate (see Materials and Methods). Panels (**c**) and (**d**) show the current as acquired, before subtracting the baseline. Overlaid red and blue lines show the average values.

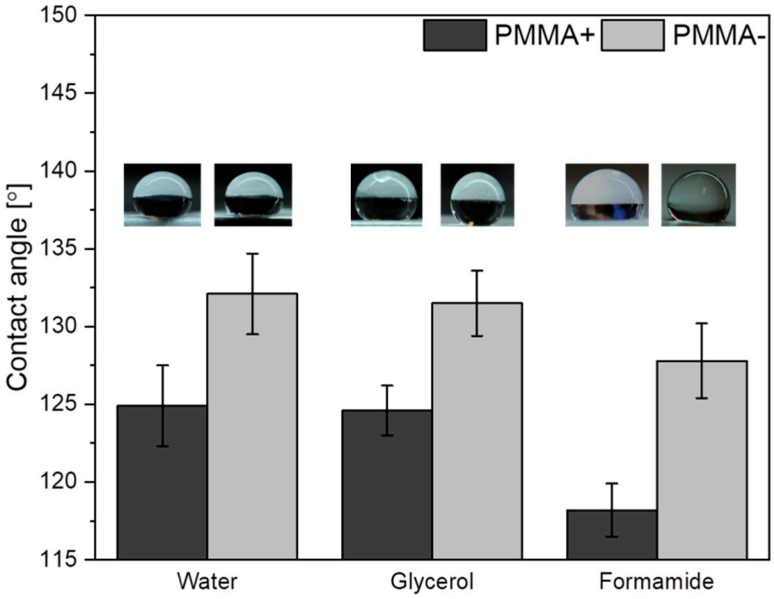

Figure S2. Contact angle and representative images of water, glycerol and formamide droplets deposited on random PMMA+ and PMMA- membranes.

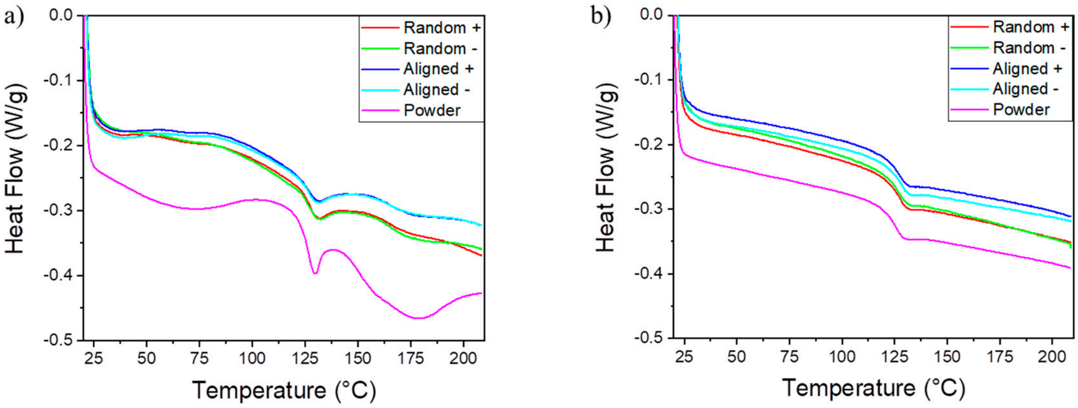

Figure S3. Differential scanning calorimetry (DSC) curves for electrospun poly methyl methacrylate (PMMA) fibers random and aligned, produced with positive and negative voltage polarity and powder at (**a**) the first and (**b**) the second heating runs.

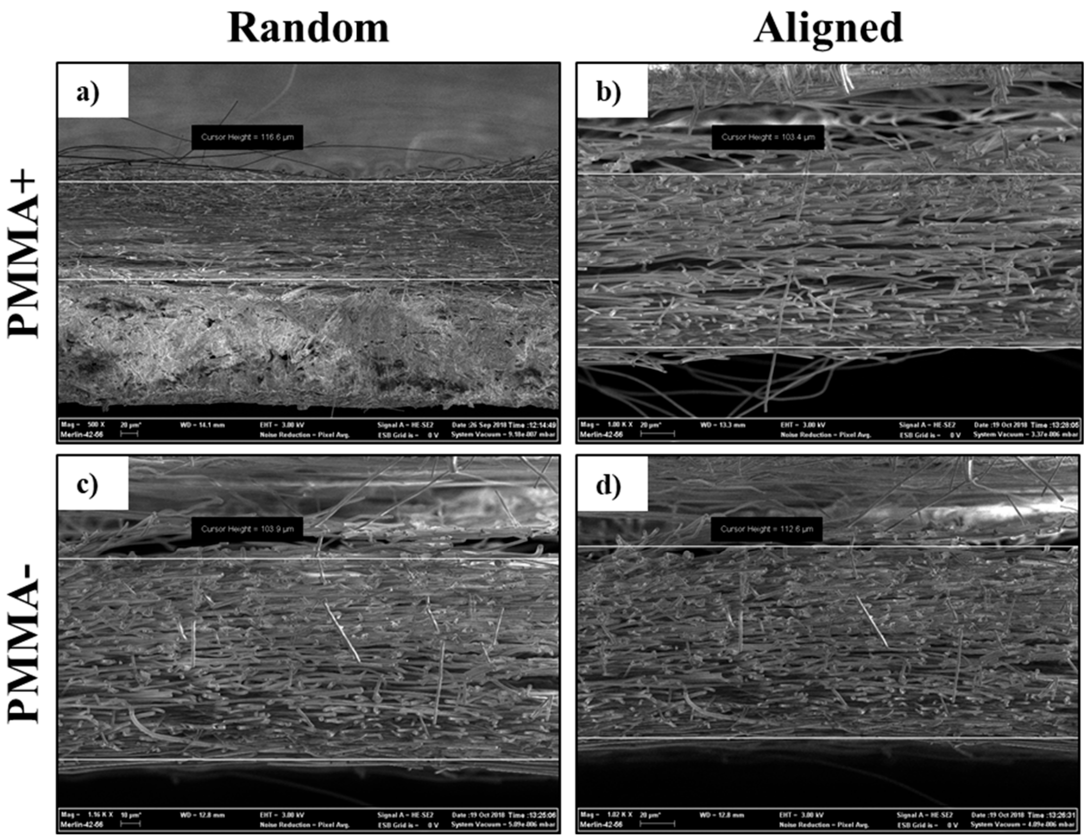

Figure S4. Scanning electron microscopy (SEM) micrographs of cross-sectional electrospun mats used for mechanical testing to measure the sample thickness of random and aligned PMMA fibers produced with (**a**), (**b**) positive and (**c**), (**d**) negative voltage polarity.

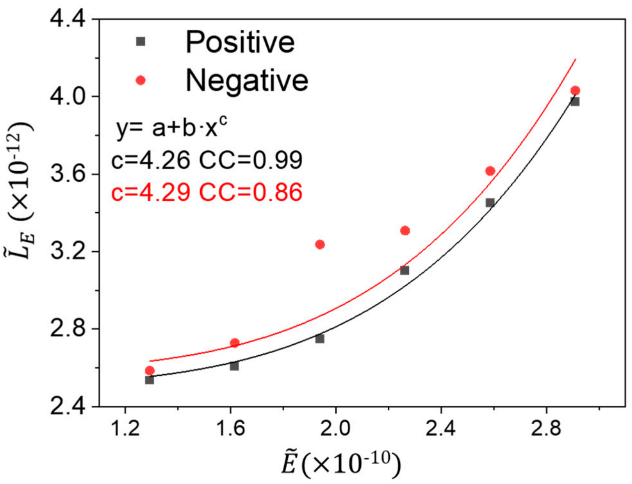

Figure S5. The dimensionless length, $\tilde{L}_E$ of the jet's straight section versus the dimensionless electrical field.

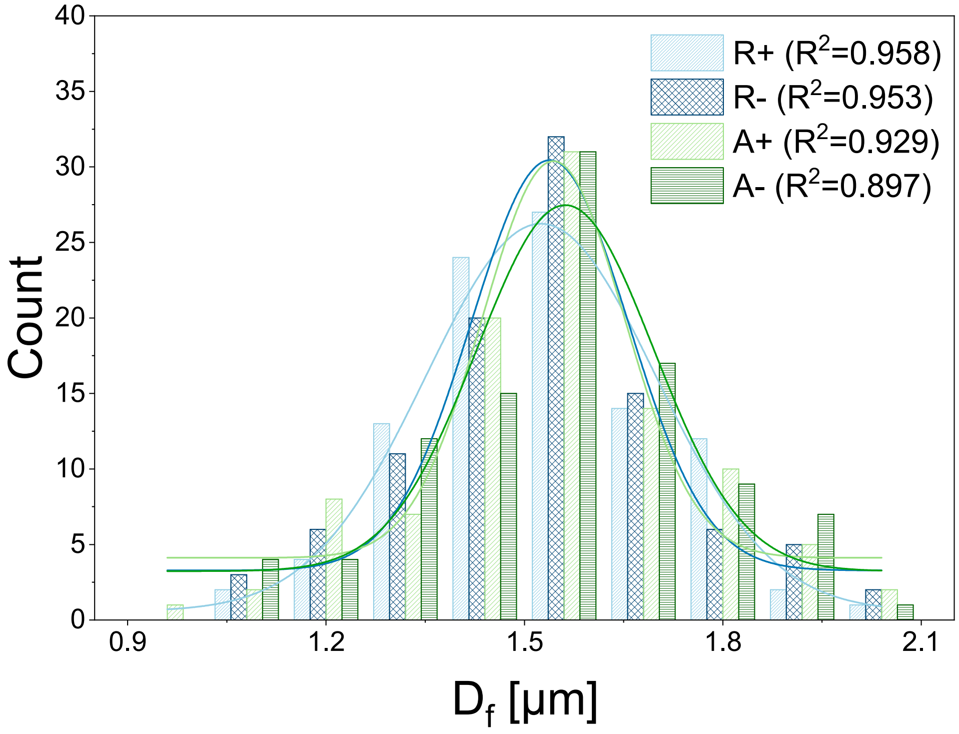

Figure S6. The fiber diameter Size distribution and fitting distributions line in a Gaussian model for PMMA samples.

Table S1. Parameters used for calculations of $\tilde{L}_E, \tilde{R}_E, \tilde{E}$ and q. ** value taken from [1].

Applied Voltage [kV]	Viscosity η [mPa·s]	Surface tension γ 10^{-3} [N·m^{-1}]	Electrical conductivity K [Ω^{-1}·m^{-1}]	Dielectric constant ε	Diameter of the jet, d [μm]
8, 10, 12, 14, 16, 18	1000**	37.10**	$3\cdot10^{-4}$**	37.50**	26 (PMMA+) 30 (PMMA-)

Table S2. Average values and standard deviation of needle voltage and current on the collector.

Type of Collector	Voltage Polarity	Needle Voltage [kV]	Collector Current* [nA]
Brass	Positive	12.5	90.3 ± 1.4
	Negative	−12.5	−86.8 ± 0.5

* After baseline subtraction. Collector current shows the average and the standard deviation over a few measurements (4 for each the positive and the negative polarities).

Table S3. Glass transition temperatures (T_g) for PMMA powder and for electrospun random and aligned fibers, produced with positive and negative voltage polarity after the first and the second heating runs. R+/− and A+/− is the random and aligned orientation of fibers produced with positive and negative voltage polarity. And the average and standard deviation values for crystallinity and molecular mass M_w.

PMMA Sample	T_g at the First Heating [°C]	T_g at the Second Heating [°C]	DSC Crystallinity [%]	M_w [g·mol^{-1}]
powder	123.9	123.8	–	–

A+	125.1	125.3	1.51 ± 0.76	291 530
A−	125.0	125.9	1.08 ± 0.20	285 600
R+	125.3	125.1	2.96 ± 1.12	289 270
R−	125.3	125.4	1.10 ± 0.35	291 950

References:

1. Shmukler, L. E., Van Thuc, N., Safonova, L. P. *Ionics (Kiel).*, **2013**, *19*, 701–707.

© 2020 by the authors. Licensee MDPI, Basel, Switzerland. This article is an open access article distributed under the terms and conditions of the Creative Commons Attribution (CC BY) license (http://creativecommons.org/licenses/by/4.0/).
